# Method for Improving Range Resolution of Indoor FMCW Radar Systems Using DNN

**DOI:** 10.3390/s22218461

**Published:** 2022-11-03

**Authors:** Hwesoo Park, Minji Kim, Yunho Jung, Seongjoo Lee

**Affiliations:** 1 Department of Information and Communication Engineering, Sejong University, Seoul 05006, Korea; 2 Department of Convergence Engineering of Intelligent Drone, Sejong University, Seoul 05006, Korea; 3 Department of Smart Drone Convergence, Korea Aerospace University, Goyang 10540, Korea; 4 School of Electronics and Information Engineering, Korea Aerospace University, Goyang 10540, Korea

**Keywords:** frequency modulated continuous wave RADAR, deep neural network, OS-CFAR, root mean square error

## Abstract

Various studies on object detection are being conducted, and in this regard, research on frequency-modulated continuous wave (FMCW) RADAR is being actively conducted. FMCW RADAR requires high-distance resolution to accurately detect objects. However, if the distance resolution is high, a high-modulation bandwidth is required, which has a prohibitively high cost. To address this issue, we propose a two-step algorithm to detect the location of an object through DNN using many low-cost FMCW RADARs. The algorithm first infers the sector by measuring the distance to the object for each FMCW RADAR and then measures the position through the grid according to the inferred sector. This improves the distance resolution beyond the modulation bandwidth. Additionally, to detect multiple targets, we propose a Gaussian filter. Multiple targets are detected through an ordered-statistic constant false-alarm rate (OS-CFAR), and there is an 11% probability that multiple targets cannot be detected. In the lattice structure proposed in this paper, the performance of the proposed algorithm compared to those in existing works was confirmed with respect to the cost function. The difference in performance versus complexity was also confirmed when the proposed algorithm had the same complexity and the same performance, and it was confirmed that there was a performance improvement of up to five-fold compared to those in previous papers. In addition, multi-target detection was shown in this paper. Through MATLAB simulation and actual measurement on a single target, RMSEs were 0.3542 and 0.41002 m, respectively, and through MATLAB simulation and actual measurement on multiple targets, RMSEs were confirmed to be 0.548265 and 0.762542 m, respectively. Through this, it was confirmed that this algorithm works in real RADAR.

## 1. Introduction

RADAR can effectively detect objects in various weather conditions or external environments compared to other sensors such as cameras and LiDAR [1,2,3]. In addition, RADAR uses millimeter waves to detect information on objects, so it can use miniature and lightweight antennas and transceivers and operate with low power consumption [4,5]. For this reason, RADAR can be effectively used in a variety of fields that require object detection [6,7].

RADAR can be divided into continuous-wave (CW) RADAR and pulse RADAR, according to the transmission/reception signal of the RADAR. First, pulse RADAR transmits radio waves in the form of pulses in RADAR transmission and reception, and then receives incoming signals from the target during the time between pulses. Although it has a high-range resolution for position measurement, it can incur the problem of the frequency band overlapping with the existing system because of an excessively wide frequency band; there is also the problem that a large-sized is used [8,9]. CW RADAR can detect moving objects by use of continuous waves, and it can also detect the speed of objects. However, it is not possible to know exactly where an object is located because the distance information cannot be known [10,11].

Frequency-modulated continuous wave (FMCW) RADARs compensate for the shortcomings of CW RADARs. FMCW RADAR has the advantages of being able to detect object distance, having a modulating bandwidth of up to 1 GHz, and being able to detect multiple objects. For these reasons, FMCW RADAR is suitable for monitoring indoor objects. Further details on the RADARs can be found in Emanuele et al. [12].

(1) $$\hat{r}=\frac{c}{2B}$$

Range resolution refers to the extent to which FMCW RADAR can measure objects in more detail. Range resolution is calculated as shown in Equation (1), where $\hat{r}$ is the range resolution, *c* is the speed of light, and *B* is the bandwidth of the FMCW RADAR. As this shows, the range resolution of the FMCW RADAR varies according to the bandwidth [13].

To detect various situations occurring indoors, a sensor capable of more detailed measurement is needed. Therefore, the range resolution value of the FMCW RADAR should be small (i.e., to achieve more-detailed measurement). However, using FMCW RADAR with small-range resolution values requires a large modulation bandwidth, which is not suitable for use as an indoor measurement sensor because it is expensive. Currently, algorithms have been developed to measure indoor conditions using relatively inexpensive passive infrared (PIR) sensors, LEDs, and photodiodes [14]. However, since PIR sensors detect temperature changes using infrared rays, they are not measured if they are not moved or moved finely, and algorithms that detect using LEDs and photodiodes are easily influenced by external environmental factors such as light [15]. These considerations have increased interest in the use of RADAR, which is relatively less sensitive to the external environment. Accordingly, numerous studies have been conducted to improve the range resolution using low-cost FMCW RADAR using a small bandwidth [16,17,18].

Algorithms have been developed to improve the range resolution by expanding the number of points of a signal containing bit frequency in the time domain of FMCW RADAR [19]. A typical method is to expand the number of points of a signal using mirror padding, which is a method of copying and attaching the signal after the signal. Improving the range resolution through this method has the disadvantage that estimating the distance to the object after performing a fast Fourier transform (FFT) creates main lobes and side lobes, which reduces the ability to distinguish the object. In addition to these methods, other algorithms have been developed to minimize the side lobe of the signal [20]. In the case of the adaptive mirror padding (AMP) method, the number of points of the signal is expanded by checking the pole of the beat frequency signal and copying the signal based on the location of the pole. In addition, the phase correct padding (PCP) method checks the phase of the beat frequency signal to identify the most similar part of the original signal through the slope and size of the phase at the last part of the signal and expands the signal to increase its number of points. This method has a disadvantage in that there is a limit to improving the range resolution as a method of expanding the range resolution through the information of the beat frequency signal.

Another algorithm that improves range resolution uses STFT to spectrogram a signal, extract features, and detect situations through SVM [21]. That algorithm can detect objects with high precision, but it is difficult to apply the algorithm to multiple targets. Therefore, it is not suitable for detecting various situations in indoor environments.

Recently, algorithms for improving range resolution through artificial intelligence have been developed [22,23]. Through deep learning, a time domain signal containing a beat frequency is learned and so the point of the signal is expanded to improve the range resolution. However, it is difficult to measure spatially with this method. In addition to these methods, algorithms have also been developed to measure objects more accurately through 2D DNNs. However, this approach does not increase the range resolution but reduces the estimation error of the measured object. Nevertheless, detecting multiple objects with this method is difficult. It is not suitable for detecting an indoor environment because it is difficult to detect more diverse situations or various objects in an indoor environment.

Looking at Table 1, the advantages and disadvantages of the existing studies described above can be seen. First, passive infrared (PIR) is an existing type of sensor [14]. Field light is an existing method for detection using LEDs and photodiodes [15]. Mirror padding is a study that expands signals using mirror padding [20]. AMP and PCP extend signals [20]. Breath monitoring (BM) extracts the feature of the signal after STFT (Short-Time Fourier Transform) of the signal and detects the situation through a Support Vector Machine (SVM) [21]. (Neural Networks (NNs) extend time signals through artificial intelligence [22], and finally, deep learning-2D (DL-2D) reduces the estimation error of objects measured through 2D DNNs [23]. Through this, the performance of existing studies can be confirmed.

In this paper, we use two inexpensive FMCW RADARs to build a 2D sector and grid. The difference between this paper and the papers that improved the performance through the existing DNN [24,25] is that the complexity is improved. In the proposed method, we learn the sector position data, which is the approximate position of the object, and the grid position, which is the position of the detailed object, measured by two FMCW RADARs. It learns from it and suggests ways to improve complexity. We also propose a Gaussian filter method that can separate objects when multiple objects are detected. In this study, the distance of an object is measured using two FMCW RADARs in an indoor situation of 10 m × 10 m, and the position of the object is inferred by inferring it in two steps using DNN. The use of the two-step algorithm reduces the amount of computation compared to when not used. If a grid is set at 0.5 m intervals in a space of 10 m × 10 m, if one inference is calculated for each grid, the entire reasoning must be calculated 441 times. Inferring step 2 results in a total of 50 calculations. Accordingly, the amount of computation is reduced by about nine times. This shows that complexity has improved.

The content order of this paper is as follows. Section 2 describes the basic theory for FMCW RADAR and how to estimate distances. Section 3 describes the 2D measurement method of FMCW RADAR in indoor situations. Section 4 describes how to separate multiple targets through a Gaussian filter. In Section 5, algorithm verification is performed by comparing the simulation results of the proposed technique with actual measurement results. Finally, Section 6 describes the conclusions of the study.

## 2. FMCW RADAR Fundamentals and Distance Estimation Method

FMCW RADAR can be transmitted in sawtooth and triangular waves. However, in this paper, we describe sawtooth wave FMCW because we use sawtooth wave FMCW RADAR.

**Figure 1:**
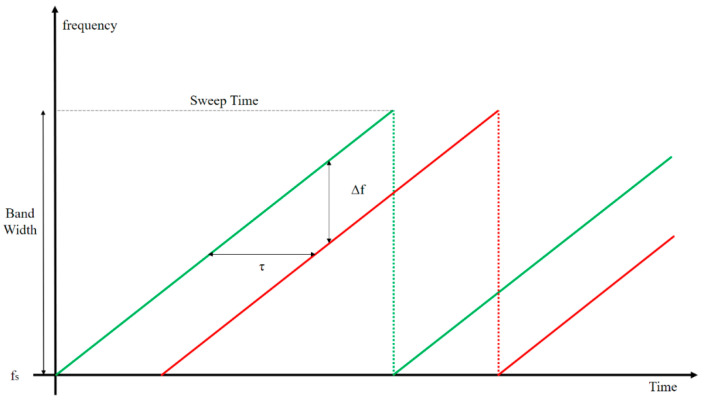
Transmit and receive FMCW RADAR signals.

Figure 1 shows the FMCW sawtooth wave. The horizontal axis of the plot is time, the vertical axis is frequency, the transmitted signal is a green line, and the signal received after hitting the stationary object is a red line. In this study, there is a frequency difference between the received signal and the transmitted signal due to a delay time, which we refer to as beat frequency.

Equation (2) is a formula for distance. The time delay of the transmission signal and the reception signal entails the difference in distance from the object measured in FMCW RADAR, which can be obtained through beat frequency. Here, sweep time and bandwidth are unique parameters of the FMCW RADAR and are consequently constant values. ***c*** is the speed of light, Δ*f* is the frequency difference between the transmit and receive waveforms, and τ is the temporal difference between the transmit and receive waveforms.

(2) $$r=\frac{\tau \cdot c}{2}=\frac{c\cdot \Delta f\cdot Sweep\;Time}{2\cdot Band\;Width}$$

**Figure 2:**
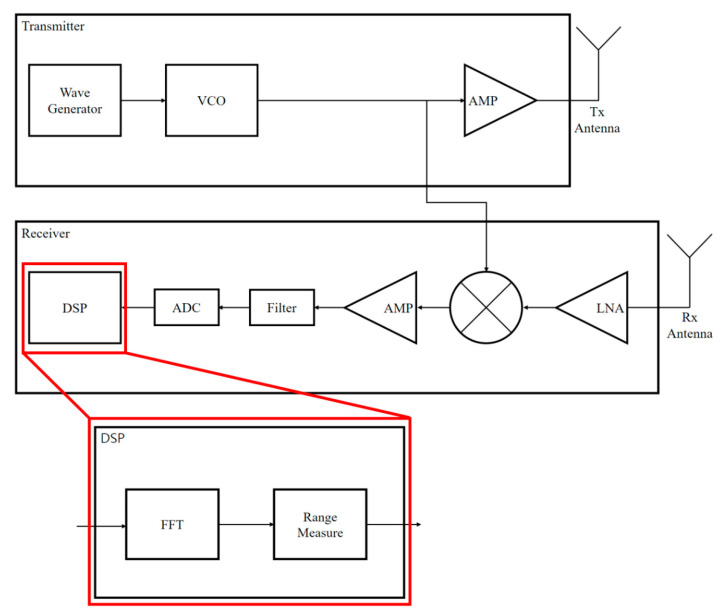
FMCW RADAR Structure.

The FMCW RADAR structure is shown in Figure 2. First, the wave generator generates a constant linear transmission waveform through the PLL. Thereafter, the frequency of the transmission waveform is modulated through the VCO, amplified by the AMP, and transmitted through the Tx Antenna. Then, the transmitted signal hits the target and is reflected, and the reflected signal is received by the Rx Antenna. The received signal is amplified through a low noise amplifier (LNA), and then a mixer is multiplied to obtain beat frequency, and then a filter is used to extract beat frequency. The extracted beat frequency is converted into a digital signal, and then DSP processing is performed. In this study, the DSP process converts beat frequency into frequency domain through FFT and then measures the distance.

The measurement distance of the FMCW RADAR can be confirmed by the method used by Seongmin, et al. [20], and the channel information for the corresponding FMCW RADAR can be confirmed through that in Chang-Heng, et al. [26] and Peli, et al. [27].

## 3. Two-Dimensional Measurement Method of FMCW RADAR in Indoor Situations

**Figure 3:**
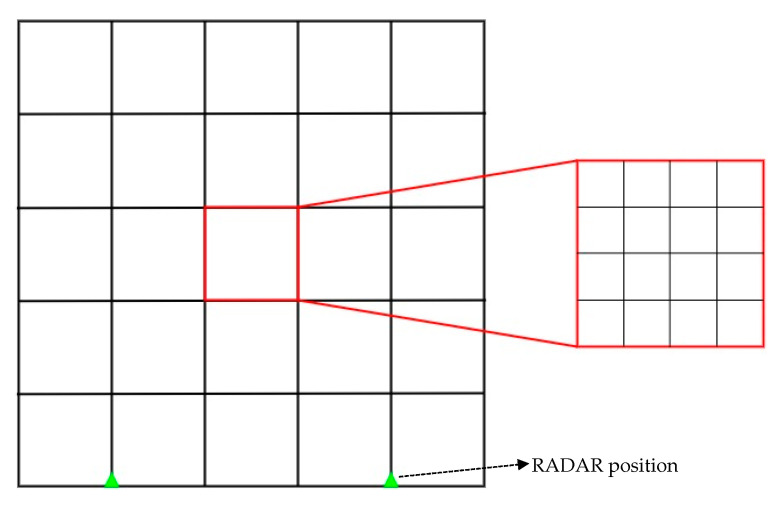
Describe the valid target positions of the indoor environments.

In this paper, two FMCW RADARs are used to detect indoor situations. Here, the indoor situation refers to a space of 10 m × 10 m. Through Figure 3, the valid (distinguishable) positions of targets set in this paper can be seen in indoor environments. In the indoor environment, sectors were divided at 2-mm intervals, and as a result, 25 sectors were divided. In one sector, grids were placed again at intervals of 0.5 m, resulting in 25 grids. In this study, the FMCW RADAR exists on the left and right sides, respectively, and the location of the FMCW RADAR can be confirmed by a triangle display. The indoor space was assumed to be 10 m × 10 m, and it can be applied even in a larger size. Accordingly, the number of sectors may be variably applied. In addition, the distance difference between RADARs can be as wide as the maximum indoor space, and it should be set at a minimum by the distance interval of wave length/2 of the millimeter-wave MIMO RADAR, which has approximately 6.2-mm intervals. However, in this paper, the interval between FMCW RADAR was measured by dropping 6 m for convenience to identify the pattern. The wavelength can be expressed by Equation (3), where *λ* is the wavelength, *c* is the speed of light, and *f* is the center frequency, 24 GHz.

(3) $$\lambda =\frac{c}{f}$$

**Figure 4:**
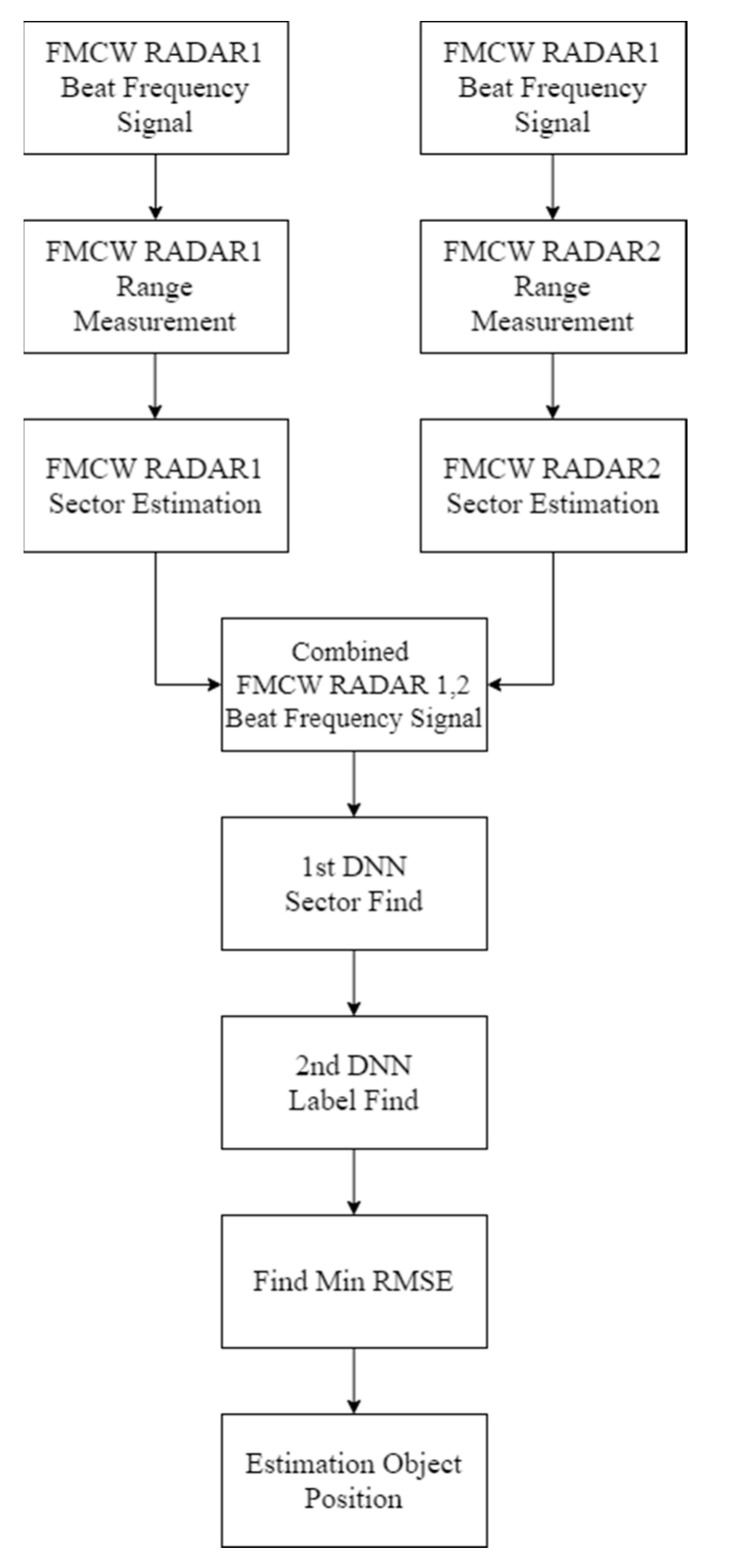
Algorithmic description diagram.

Figure 4 shows the object estimation algorithm. Two FMCW RADARs are used to detect objects in the corresponding indoor environment. Objects are not measured simultaneously using two FMCW RADARs, but one FMCW RADAR. The distance information measured by each FMCW RADAR is then integrated. Thereafter, the integrated distance information is inferred through the DNN to estimate the sector. In the estimated sector, the inference is performed through DNN to confirm the results. After inference in each sector, RMSE is measured, and the smallest value is considered the correct answer.

**Figure 5:**
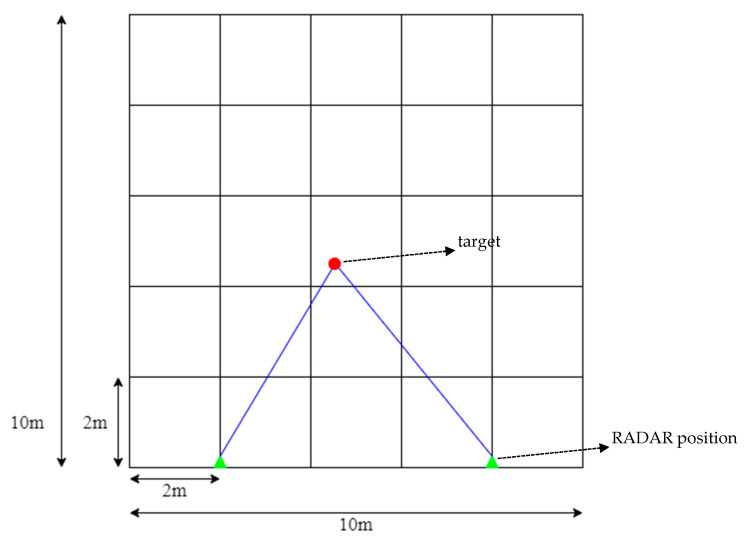
Sector Find though DNN.

**Figure 6:**
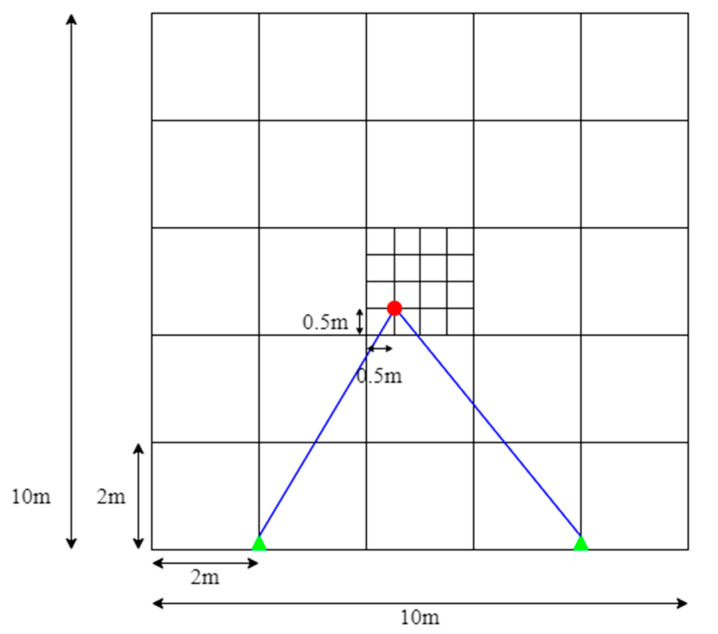
Label Find through DNN.

Figure 5 shows the Sector Find method using DNN. As mentioned earlier, the sector is a space of 2 m × 2 m, and there are twenty-five sectors in an indoor environment of 10 m × 10 m. Thereafter, grids exist at 0.5-m intervals in each sector. In this study, the inference is largely started by one sector to find the sector. After that, using the method shown in Figure 6, detailed labels are found within each sector. This approach uses the same DNN structure for inference.

In this paper, to reduce complexity, DNN inference is performed in two steps, as follows, and the computation amount is reduced compared to detecting the entire existing area. Table 2 shows that the existing entire area uses 441 labels, and the proposed algorithm only uses 25 labels twice. As a result, there is a nine-fold difference in calculation volume.

Table 3 shows the difference in RMSE between the entire-area-finding method (one-step DNN) and the two-step DNN method. RMSE was calculated with 100 repetitions. In this study, in the case of the two-step DNN, if the wrong sector is inferred in the first step, the second step is inferred to find the label, and the difference between the inferred result label and the actual location of the label is obtained. As a result, about 0.3823 m is deduced from the entire area and about 0.3542 m for two steps.

## 4. Separate Multiple Targets through Gaussian Filters

**Figure 7:**
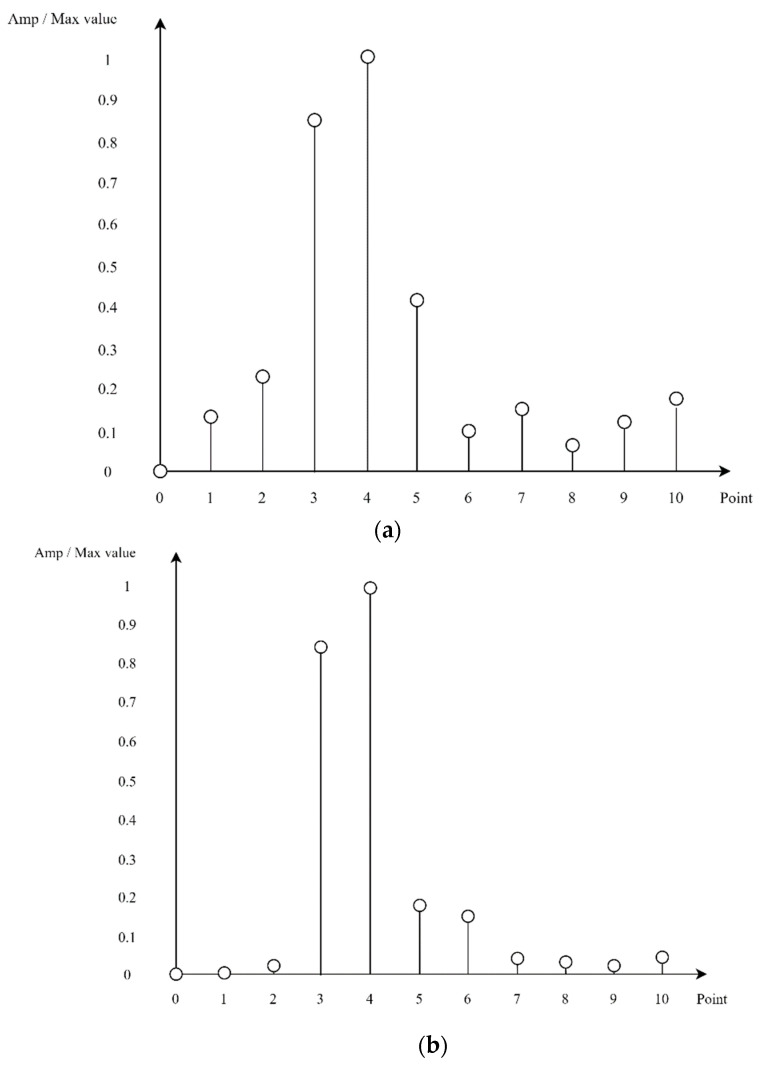
Frequency-domain signals for single target (**a**) and two targets (**b**). (**a**) Case of there is object at 3.5 m; (**b**) Case of there are objects at 3 and 4 m.

**Figure 8:**
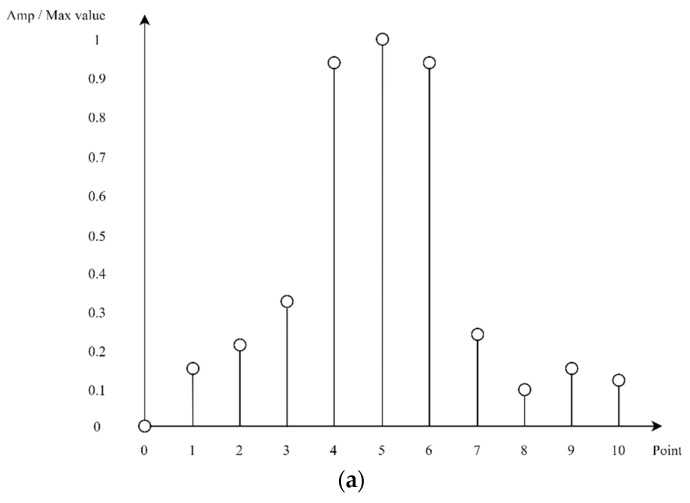

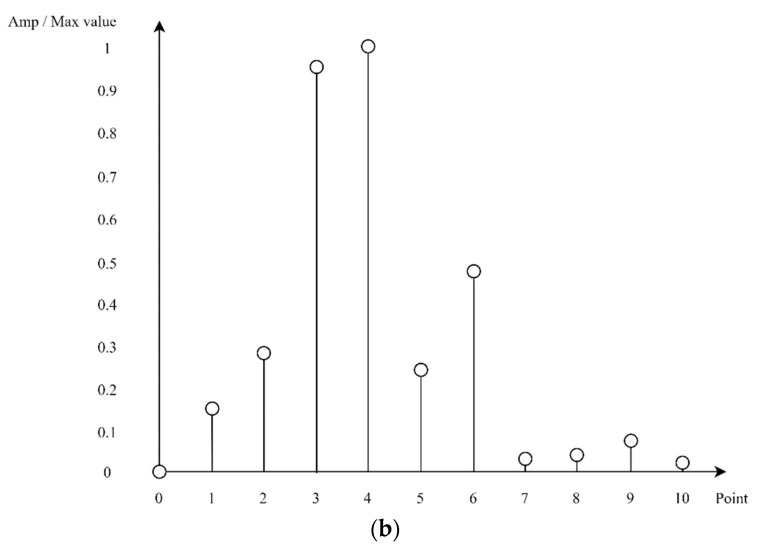
Frequency-domain signals for multiple targets. (**a**) Case of locations of targets are ambiguous; (**b**) Case of positions of the two objects are between 3 and 4 m and between 5 and 6 m.

**Figure 9:**
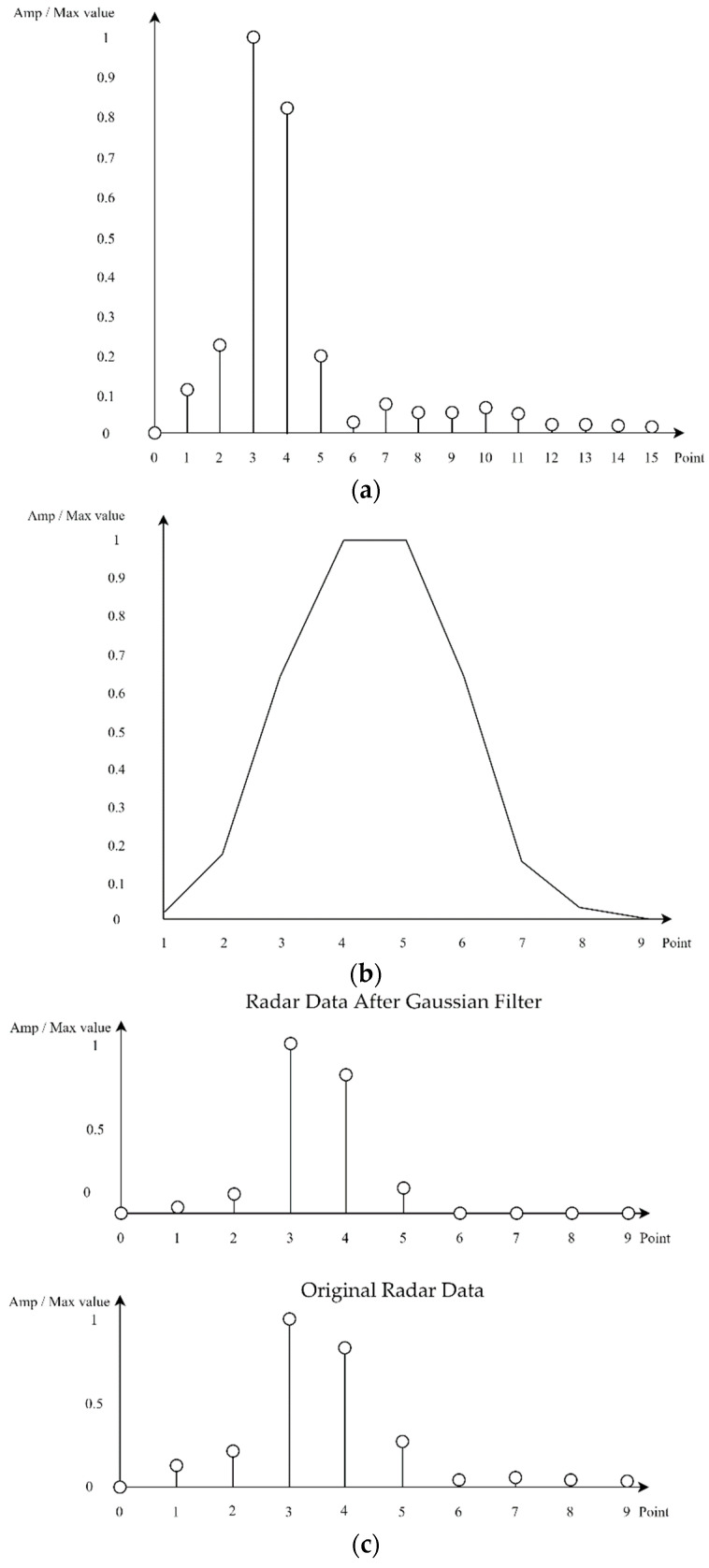
Frequency Domain Signal for the result of isolating Gaussian filters and multiple targets. (**a**) Frequency-domain RADAR signals for two targets; (**b**) Frequency response characteristics of Gaussian filter; (**c**) Comparison between a single target extracted through a Gaussian filter from multiple targets and a single target measured through a RADAR simulation.

In this paper, when there are multiple targets, we separate them and infer them through DNNs. In this case, there may be a situation where it is unknown whether the two objects are actually one object. In this study, in Figure 7, Figure 8 and Figure 9, the y-axis is the magnitude of the signal, which is the magnitude of the frequency signal, which can be seen as the amplitude of the signal. In this study, the magnitude of the corresponding signal is expressed by normalization with the max value. The x-axis is the FFT Point and starts from 0. As can be seen from Figure 7, if the range resolution is 1 m, it is difficult to see the difference between Figure 7a, where a 3.5-m object is detected, and Figure 7b, where objects are detected at 3 and 4 m, through the frequency domain signal. Therefore, when there are multiple targets, they can be separated according to the performance of the range resolution to separate multiple targets from the corresponding FMCW RADAR. As a result, each target can be detected separately according to the range resolution of the unique FMCW RADAR that detects the object, which must be twice the distance of the range resolution to detect each object separately.

In this algorithm, when the distance between targets is less than twice the range resolution, it is difficult to separate each target and infer DNN through the pattern of the corresponding signal. As illustrated in Figure 7, if a range resolution is 1 m and Figure 8a shows a peak value at 5 m and any value is measured at 4 and 6 m, it is difficult to identify how many targets exist and to separate each target and infer to DNN. In addition, Figure 8b shows that when the range resolution is 1 m, the targets are between 3 and 4 m and between 5 and 6 m. Although this is not known exactly, they are separable and can be separated to proceed with inference to the DNN.

In this study, if each target is twice the range resolution, it is necessary to utilize the pattern for the signal and reduce the remaining values. Therefore, we propose a Gaussian filter. The Gaussian filter uses the Gaussian distribution, which originally uses the Gaussian distribution so that the total sum is 1, but in this paper, the peak value is adjusted to be 1 for inferring the pattern. In addition, when using a Gaussian filter, each has a peak value of 1 for the larger of the max and max values of the pattern, and the influence on the remaining values is reduced according to the Gaussian distribution. This is used to detect an object more accurately by extracting the main lobe feature of an object using a Gaussian filter. As mentioned above, in a Gaussian filter, only the main lobe can be extracted by pressing the side lobe value. The corresponding Gaussian filter was designed using the MATLAB model and set up to accurately measure the FMCW RADAR. Many objects within the range resolution cannot be detected, and as a result, objects that cannot be measured by low-cost FMCW RADARs cannot be detected. Thus, when three or more multiple objects enter, they cannot be separated by the side lobes of the first and second closest objects. In addition, when the distance between objects is very long, it is difficult to separate the signals because the magnitude of the signal measured by the RADAR is different. This can be seen in more detail in Figure 9. In this study, when two targets are generated as shown in Figure 9a, a Gaussian filter that fits the corresponding pattern as shown in Figure 9b is generated. As can be seen in Figure 9c, the target is separated through a Gaussian filter for the target whose distance is the closest to RADAR and is compared with the frequency domain signal of a single target at the same distance measured by FMCW RADAR.

**Figure 10:**
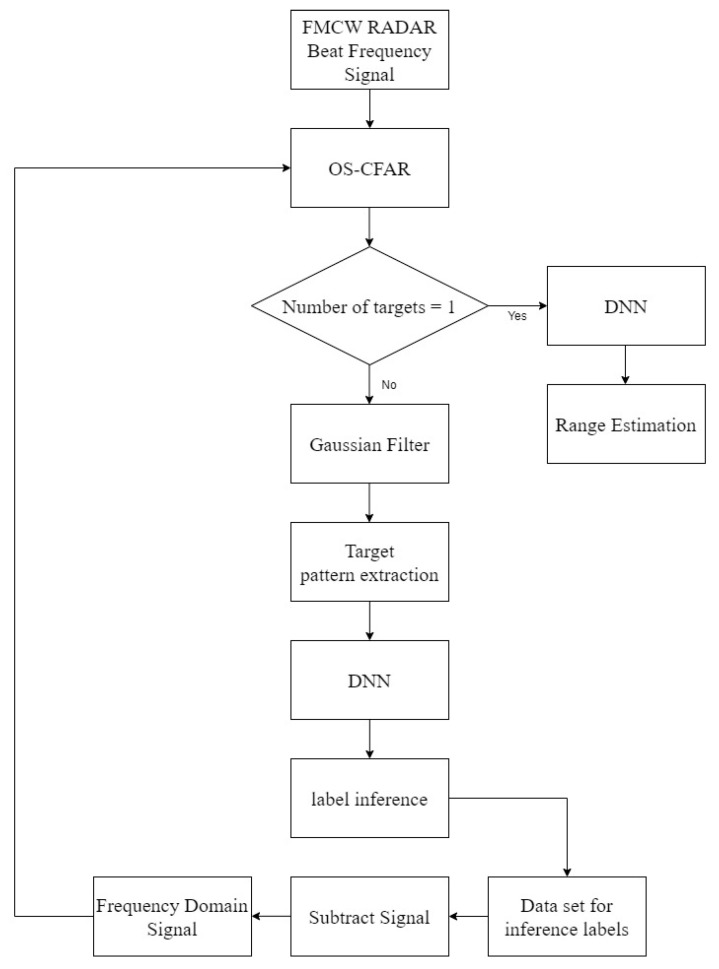
Algorithmic flowchart for multiple targets.

In the proposed algorithm, it is necessary to check whether the corresponding pattern is a multiple target or a single target. Therefore, in this paper, to identify multiple targets, we use the constant false alarm rate (CFAR) algorithm [28,29,30,31], which is used to check whether an object has been detected. The CFAR algorithm sets the false alarm rate, measures various fluctuating noises and distortions in average clutter power, and then multiplies the false alarm rate by a corresponding coefficient to variably adjust the threshold to have a fixed false alarm rate. In this case, the threshold value and the magnitude of the frequency domain signal are compared, and if the signal size is greater than the threshold value, the target may be determined; if it is small, multiple targets may be detected by determining that the target is not. The corresponding CFAR algorithms are typically order-statistic (OS) CFAR and cell-averaging (CA) CFAR, and in this paper, OS-CFAR is used to detect multiple targets. The algorithmic flow chart for separating multiple targets can be confirmed in Figure 10, and through this, multiple targets can be separated; as a result, multiple targets can be detected.

When multiple targets are separated using OS-CFAR, the objects measured in each sector and lattice do not fit the range resolution. Therefore, there is a difference in the actual distance between the object and the FMCW RADAR and the distance measured by the FMCW RADAR. Thus, the target was detected in OS-CFAR through the difference between the actual distance difference in units of 1 m and the peak value of the measured distance through OS-CFAR, and objects that could not be detected were excluded. In addition, in multi-target cases, if peak points are detected in OS-CFAR by a difference of 2 m, the objects are measured as multiple targets.

In this study, when multiple targets are separated using OS-CFAR, there is a probability of failure of detection of OS-CFAR itself. OS-CFAR was confirmed by Seongmin, et al. [20], and False Alarm Rate = 10^−5^, Training Cell = 32, Guard Cell = 2, and Rank = 24 were set. The result of checking OS-CFAR through the proposed algorithm. The false alarm rate for a single target is 8.64%, and the false alarm rate for multiple targets is 11.82%.

## 5. Experimental Results

In this paper, the performance of the proposed algorithm was verified using the MATLAB tool and FMCW RADAR.

### 5.1. MATLAB Simulation

**Figure 11:**
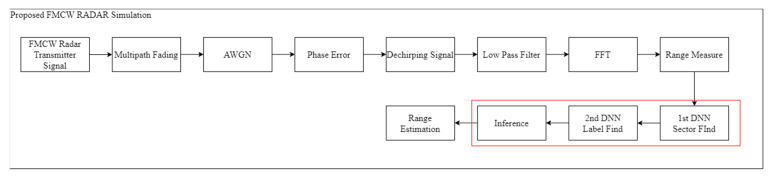
MATLAB Simulation Block Diagram. (Red box: the module proposed in this work).

Figure 11 shows the MATLAB simulation block diagram. In this study, multipath fading, phase error at 24 GHz, and AWGN were added to implement a simulation environment similar to the actual situation. Through Table 4, simulation parameters were prepared by referring to the parameters of the actual FMCW RADAR.

**Figure 12:**
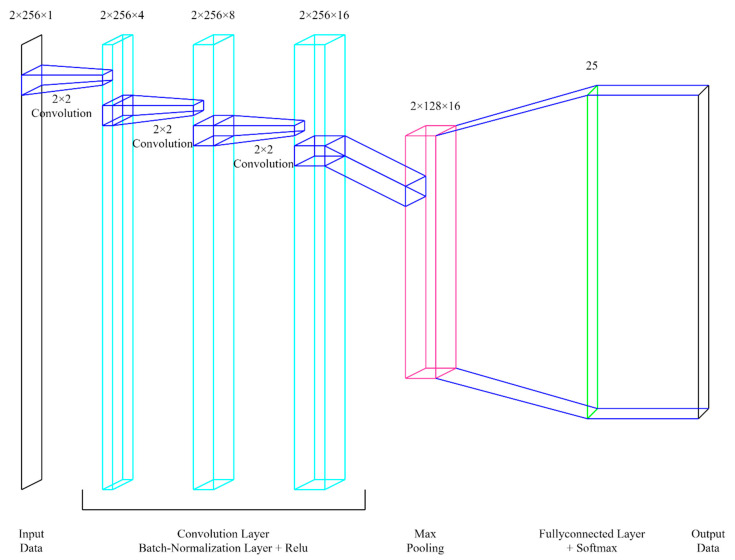
DNN structure.

**Figure 13:**
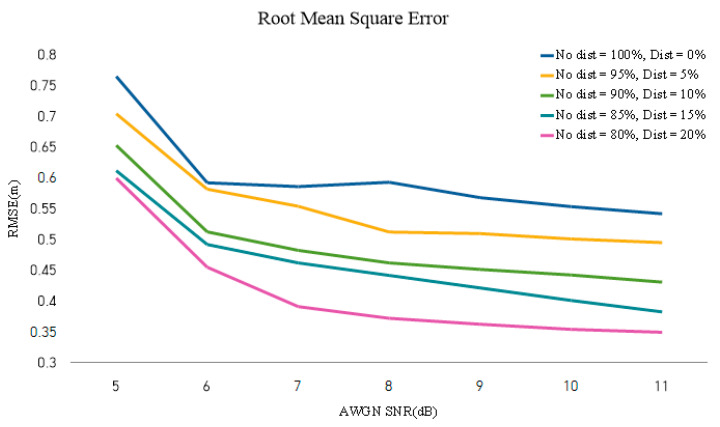
RMSE by AWGN SNR according to distorted data content of learning data.

MATLAB simulations for DNNs are performed as shown in Figure 12. In this study, FMCW RADAR’s frequency-domain signal extracted through MATLAB is used for DNN learning. The performance is then measured through distance differences (i.e., through RMSE, according to the inferred label of DATA and the label of the corresponding DATA). In this study, the corresponding signal uses randomly opened DATA in each grid and first learns with multipath, AWGN, and phase error-free data to check the results. Subsequently, the results of learning all distortion data and no distortion are added, and the results of the FMCW RADAR output for 5 dB, 6 dB, 7 dB, 8 dB, 9 dB, and 10 dB are combined, respectively. In this study, the performance is checked by mixing 5%, 10%, 15%, and 20% of data with distortion in DATA without distortion, respectively. This is confirmed in Figure 13. Assuming that there is data in the grid, the corresponding result outputs test data every 5 dB, 6 dB, 7 dB, 8 dB, 9 dB, and 10 dB, and then uses the data to conduct inference. In this study, the RMSE is measured, and as a result, it can be confirmed that the data mixed with 20% have the best performance. Measurement below 4 dB is not possible, because the data are broken.

In Figure 12, the input is a 2 × 256 × 1 matrix. This is because the measured results from the two FMCW RADARs are transformed into a matrix through an FFT transformation of 256 points each. DNN datasets were generated using MATLAB tools and examples, and datasets for each sector were prepared separately. The x- and y-axes are each made to have a distance of −0.2 m to 0.2 m from the labels per label inside each sector, and 110 per label are independently randomized. In this study, 100 data points were used as test data and 10 were used as inference data for performance verification.

Performance is measured by comparing the DNN structures proposed in previous studies with those proposed in this paper. In this study, since the existing papers do not include an algorithm to divide the sector, it is assumed that a 0.5-m lattice structure exists in the entire grid structure of 10 m × 10 m excluding the corresponding part, and the result is verified by simulation with MATLAB. In this study, the 1D DNN structure simulates the DNN structure described in the paper [23] with MATLAB to confirm the results with the dataset for the DNN structure proposed in this paper. Then, the results were confirmed in the DNN structure of measuring the moving person in the room in the paper [32] under the same environment. In Table 5, results show that RMSE values of papers [23] and [32] have a performance of 0.515734 and 0.48262 m, respectively, and it can be said that the performance of the DNN structure of the proposed paper is better in the corresponding environment. In the C3 DNN structure, the convolution operation is performed three times.

#### 5.1.1. Single Target

**Figure 14:**
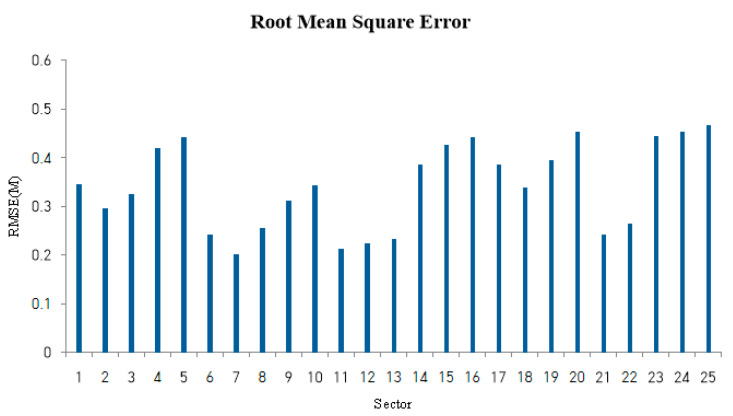
RMSE value for inference to a single target per sector.

Fifteen random data points per grid are extracted from a single target, and 100 learning data points are extracted to proceed with inference. In this case, the ideal indoor situation is assumed to be AWGN SNR 10 dB and simulation is performed. We measure the RMSE in each sector and check the results. In this study, the RMSE is measured 20 times per sector and calculated as the average of these values. This can be confirmed in Figure 14.

The average of the measured RMSE values in each sector was obtained, confirming that this algorithm has an RMSE value of 0.2242 m in a single target. In addition, the RMSE value changes depending on the sector, and first, in this simulation, the error is severe for objects within 2 m, and in general, the object on the right has a higher RMSE than the object on the left. The type is output like the localization error discussed in the paper [23].

#### 5.1.2. Multi Target

To detect multiple targets, when measuring the distance to the object in the following two FMCW RADARs, we find targets that each are more than 2 m apart. In this study, for simulation, the corresponding objects were found for two objects, which are set as shown in Table 6.

To detect multiple targets, OS-CFAR was applied to apply a Gaussian filter to the corresponding five objects. In addition, we extract close targets and subtract 100 datasets for the sector and label the corresponding objects from the original signal that detected two objects after DNN inference, respectively. The power value is measured to extract the signal that becomes the lowest power value. This signal is then inferred back to the DNN to confirm the result. In this study, each of the above five targets was repeated 15 times, and the results are shown in Table 7. In this case, the RMSE is obtained as an average of the difference in distance between the inference value of each target and the actual position. In this study, the value of the RMSE for each case can be checked.

In the simulation, the RMSE value for inference is output like the RMSE of a single target, because the value for the main lobe is alive when the measurement is performed by applying a Gaussian filter to the first target. For the second target, we subtract the dataset for the first target from the signal measured by the multiple targets and obtain the dataset for the second target with the lowest power value. Again, we check the inference in the DNN. In this study, a value corresponding to the main lobe of the second target may be eliminated due to distortion, such as multipath fading and AWGN of the first target. Thus, the inference result of the second target is output larger than the inference result of the first target.

### 5.2. Actual Measurement

**Figure 15:**
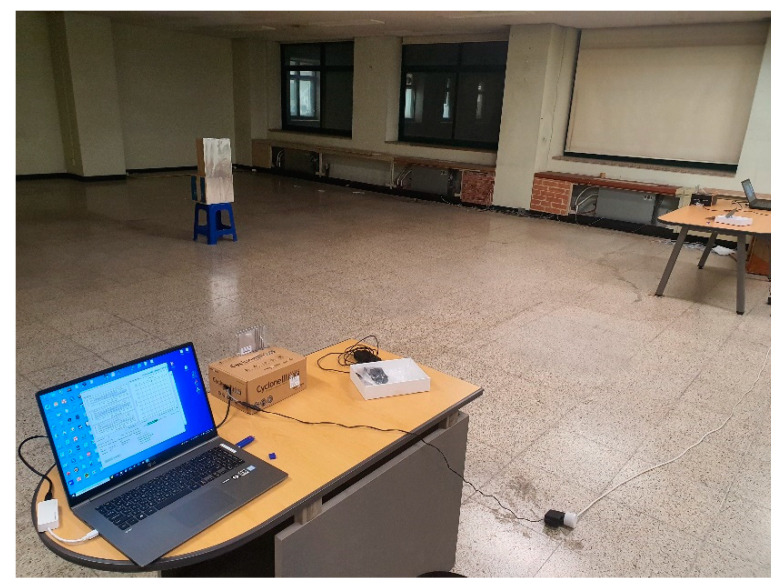
Actual measurement environment.

**Figure 16:**
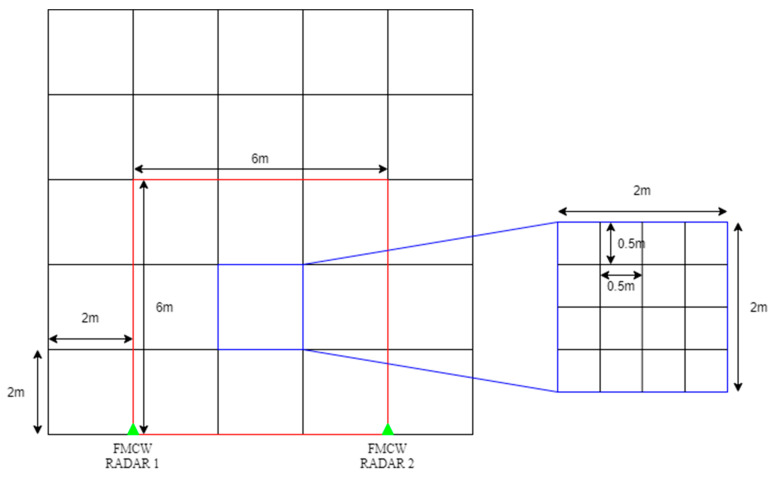
Actual measurement area.

The actual measurement was conducted in an empty classroom at Sejong University. Due to the space limitations of the classroom, the actual measurement was conducted in a space of 6 m × 6 m. In this study, the actual measurement was performed at 6-m intervals between RADARs, and the measurement was performed as shown in Figure 15. Figure 16 shows the actual measured area. The area where objects can be measured is the red area, and the FMCW RADAR is identified with a blue triangle. This study used a commercial FMCW RADAR, and Table 8 shows the parameters for the measurement. In this study, the reason for limiting the area where objects were measured is that the space that could be measured by walls and radiators was limited in an empty classroom without obstacles. Therefore, the actual measurement space was 6 m × 6 m.

In the actual measurement, as shown in Table 9, the RMSE is measured as higher than the simulation result. This is because we learned the FMCW simulation results in experiments on the proposed algorithm. Therefore, when actual measurements are made using the proposed algorithm, the output is worse than expected.

#### 5.2.1. Single Target

A single target was measured by selecting three points from among the areas that can be measured: the 7th grid of Sector 8, the 19th grid of Sector 12, and the 8th grid of Sector 13. Actual measurements of these grids are conducted and confirmed through DNN. This can be seen in Table 6. The results are as follows. In this study, the corresponding RMSE result value is a value obtained by repeating 100 times and averaging the differences in distance between the inference value and the actual value. Case 1 is the target of the seventh grid of Sector 8, and Case 2 is the target of the 19th grid of Sector 12. Case 3 is the target on the eighth grid of Sector 13. The average RMSE of the corresponding targets was measured to be 0.41002 m.

#### 5.2.2. Multi Target

Actual measurements were performed on several targets in an area that can be measured. Since the distance to the target measured in each FMCW RADAR should differ by 2 m or more, the actual measurement was conducted on two objects. Afterward, the RMSE is measured to confirm the performance. The corresponding RMSE has objects in the third and 18th grids of Sector 8 and Sector 13 and extracts 100 data for the objects and obtains them as the average of the distance differences between the inferred and actual grids. In this case, the target of the No. 8 Sector is 0.55 m, and the target of the No. 13 Sector is 0.975084 m. In this study, the average RMSE of the two targets is 0.762542 m.

#### 5.2.3. Performance Comparison with Existing Studies

To this end, the performances of the DNN structure proposed in this paper and the DNN structure of previous studies were verified. As shown in Table 10 below, the RMSE performance in this paper is compared with the previous study described in Section 5.1.1. In this case, performance verification is performed on a single target. In this case, the RMSE is the average distance difference between the inferred value and the actual value by repeating the case of Label 5 of Sector 8 100 times. In this study, in Case 1 the RMSE is the same as in the proposed paper, and in Case 2 the complexity is the same as in the proposed algorithm. In Table 10, *r*1 and *r*2 represent the ratio of RMSE and computational complexity of the existing algorithm to the proposed algorithm. At this point, the complexity is determined by the number of convolutions. Finally, the cost function is determined as the square root of the product of *r*1 (ratio of RMSE) and *r*2 (ratio of computational complexity). As the complexity of the DNN increases, the object detection performance increases. Therefore, the performance index was used to express the performance versus computational complexity of the DNN, which is expressed as a cost function, as shown in Table 10.

## 6. Conclusions

In this paper, we propose a two-step algorithm, an algorithm inferred from DNN using two FMCW RADARs to improve range resolution. The proposed algorithm estimates the sector, which is the approximate location of the object, by measuring the distance from the object in each FMCW RADAR. After estimating the sector, we measured the position of each label through the grid and the exact position of the object in the estimated sector. In this paper, the performance of the algorithm was tested in a limited space, of 10 m × 10 m, assuming an indoor situation. The performance of the algorithm was validated by using MATLAB tools to generate test datasets and validation datasets for the location of objects. In addition, after making a DNN model using the dataset, performance verification was completed with RMSE through actual FMCW RADAR. Performance was verified by separating multiple targets through a Gaussian filter, and it was confirmed that multiple targets can also be measured. The proposed algorithm has almost no performance difference from those in other papers, and it is confirmed that the amount of computation is reduced through the cost function, and there is an up-to-five-fold difference in complexity compared to the performance. This algorithm can be effectively applied to the IoT field because it can detect indoor conditions using low-cost FMCW RADAR.

## Figures and Tables

**Table 1:** Performance of existing studies.

	PIR [14]	Field Light [15]	Mirror Padding [20]	AMP and PCP [20]	BM [21]	NN [22]	DL-2D [23]
Space Search	O	O	X	X	O	X	O
Multitarget	X	X	O	O	X	O	X
Range Precision Enhancement	O	O	O	O	X	O	X
Detecting a Stationary target	X	O	O	O	O	O	O

**Table 2:** Difference between the total zone and the calculations of the proposed algorithm.

Algorithms	Computational Complexity (the Number of Labels)
whole sector finding (1-step DNN)	441
two-step DNN	50

**Table 3:** RMSE difference between the entire zone and the proposed.

Algorithms	RMSE (Meter)
whole sector finding (1-step DNN)	0.3823
2-step DNN	0.3542

**Table 4:** MATLAB simulation environment.

Parameter	Value
Bandwidth	150 MHz
Sweep Time	6.8267 us
Sampling Rate	37.50 MHz
FFT point	256
Range Resolution	1 m
Center Frequency	24 GHz
Spacing between RADARs	6 m
Size of Sector	2 m × 2 m
Number of Sectors	25
Spacing of Grid within the Sector	0.5 m
Number of Grid within the Sector	25

**Table 5:** Comparison of the proposed DNN structure with the DNN structure of the existing paper.

DNN Structure	RMSE (Meter)
1D DNN Structure	0.515734
C3 DNN Structure	0.48262
Proposed DNN Structure	0.3542

**Table 6:** Setting up Sectors and Labels for Targets.

	Target 1		Target 2	
	Sector	Label	Sector	Label
Case 1	7	4	19	20
Case 2	8	3	18	3
Case 3	8	5	18	21
Case 4	6	5	16	17
Case 5	8	25	25	1

**Table 7:** RMSE value for multiple targets.

		RMSE (Meter)
Case 1	Target 1	0.4
	Target 2	0.6154
	Total	0.5077
Case 2	Target 1	0.4
	Target 2	0.7125
	Total	0.55625
Case 3	Target 1	0.45
	Target 2	0.7251
	Total	0.58755
Case 4	Target 1	0.4
	Target 2	0.76725
	Total	0.583625
Case 5	Target 1	0.35
	Target 2	0.6624
	Total	0.5062

**Table 8:** FMCW RADAR physical characteristics.

Parameter	Value
Bandwidth	150 MHz
Sweep Time	6.8267 μs
Sampling Rate	37.50 MHz
FFT point	256
Range Resolution	1 m
Center Frequency	24 GHz

**Table 9:** Actual target measurement RMSE.

	RMSE (Meter)
Case 1	0.42652
Case 2	0.41103
Case 3	0.39251

**Table 10:** Comparison of actual measurement performance between the proposed DNN structure and the DNN structure of the existing paper.

		Proposed DNN	C3 DNN [32]		1D DNN [23]	
			Case 1	Case 2	Case 1	Case 2
RMSE	meter	0.3542	0.3621	0.7265	0.353	0.8952
	ratio (*r*1)	1	1.022	2.051	0.997	2.527
Computational Complexity	# of conv.	819,200	23,814,000	793,800	9,906,624	889,056
	ratio (*r*2)	1	29.07	0.969	12.093	1.085
Cost func ($=\sqrt{r1\times r2}$)		1	5.451	1.41	3.472	1.656
